# Dexrazoxane Protects Against Hand–Foot Syndrome–Like Skin Damage in Pegylated Liposomal Doxorubicin‐Treated Mice

**DOI:** 10.1155/jt/1358796

**Published:** 2026-01-30

**Authors:** Kentaro Nishida, Juna Tanaka, Chihiro Hashimoto, Masayuki Tanaka, Takahisa Kuga, Shogo Shigeta, Kazuyuki Kitatani

**Affiliations:** ^1^ Department of Integrative Pharmaceutical Sciences, Faculty of Pharmaceutical Sciences, Setsunan University, Hirakata, Osaka, Japan, setsunan.ac.jp; ^2^ Laboratory of Clinical Pharmacy, Faculty of Pharmaceutical Sciences, Setsunan University, Hirakata, Osaka, Japan, setsunan.ac.jp; ^3^ Laboratory of Analytics for Biomolecules, Faculty of Pharmaceutical Sciences, Setsunan University, Hirakata, Osaka, Japan, setsunan.ac.jp; ^4^ Department of Obstetrics and Gynecology, Tohoku University Graduate School of Medicine, Sendai, Miyagi, Japan, tohoku.ac.jp; ^5^ Laboratory of Biochemistry, Faculty of Pharmaceutical Sciences, Setsunan University, Hirakata, Osaka, Japan, setsunan.ac.jp

## Abstract

The anticancer drug pegylated liposomal doxorubicin (PLD) can cause hand–foot syndrome (HFS), a condition that develops in the palms and soles when pressure is frequently applied. Strategies to address HFS are insufficient. Dexrazoxane (DXZ) protects against doxorubicin‐induced cardiotoxicity, possibly via reducing topoisomerase (Topo) IIβ levels in myocytes. Previously, we developed a rat model that used three tail–vein doses of PLD to induce HFS‐like skin damage. In this study, we generated a simple mouse model of HFS‐like skin damage using rubber fastening and PLD treatment to examine potential protective effects from DXZ. Male ddY mice received PLD (16.5 mg/kg) intravenously, with the flank skin compressed by a rubber band for 48 h. DXZ (50 and 250 mg/kg) was administered intraperitoneally twice prior to PLD. Skin tissues were removed on Day 12, fixed, and stained with hematoxylin–eosin to assess epidermal thickening. Western blotting identified the expression of γH2AX (a DNA damage marker) and the doxorubicin targets Topo IIα/β. After Day 8, DXZ (both concentrations) + PLD groups exhibited less skin damage than the PLD group. The PLD group showed greater epidermal layer thickening than both the control and DXZ + PLD groups. γH2AX and Topo IIβ expression increased in the back and flank regions of PLD‐treated mice but was suppressed under DXZ + PLD treatment. Although not significant, Topo IIα expression followed an analogous pattern to Topo IIβ expression. In conclusion, we demonstrated that DXZ inhibited PLD‐induced skin damage.

## 1. Introduction

Pegylated liposomal doxorubicin (PLD) is used for treating advanced‐stage ovarian carcinoma and metastatic breast cancer. A major advantage of PLD over free doxorubicin is its lower incidence of cardiotoxicity; however, it also has the notable downside of causing hand–foot syndrome (HFS) [1, 2], also known as palmar–plantar erythrodysesthesia syndrome. This condition involves sweat‐mediated drug excretion from sweat glands, most commonly in palms and soles [3, 4], but also in the armpit and groin [5]. Repeated localized pressure and friction are hypothesized to congest blood flow and cause PLD leakage out of the capillaries, leading to high doxorubicin concentrations in affected tissues [3, 6]. Although preventive strategies such as palmoplantar cooling have been reported, effective treatment strategies for PLD‐induced HFS are still not fully understood [7].

Dexrazoxane (DXZ) has shown preventive effects against doxorubicin‐induced cardiotoxicity, and its action is thought to be functional inhibition through binding to topoisomerase (Topo) II, a target of doxorubicin [8–11]. Isoform Topo IIβ, in particular, is crucial to the cardiotoxic effects of doxorubicin, and studies on the mechanisms underlying doxorubicin‐induced cardiotoxicity and cardioprotective agents are advancing [9, 12]. Furthermore, the effect of DXZ on doxorubicin‐induced damage was most pronounced when cardiomyocytes lowly expressed Topo IIβ [13]. Therefore, DXZ‐induced downregulation of Topo IIβ in cardiomyocytes may be a mechanism underlying the observed anticardiotoxic effects.

We previously reported a rat model of PLD‐induced HFS, with skin damage on the hindpaw induced by three tail vein doses of PLD [14]. In this study, we developed a novel mouse model through a combination of one PLD dose and skin local pressurization with a rubber band. With this model, we aimed to investigate the effect of DXZ administration.

## 2. Materials and Methods

### 2.1. Experimental Animals

Male ddY mice (6 weeks old, 30–35 g; Japan SLC, Hamamatsu, Japan) were housed in cages (3–4 mice per cage) with *ad libitum* access to food and water in a controlled environment (12/12 h light/dark, ∼55% relative humidity, and 23°C). Mice were fed a normal diet (Oriental Yeast, Tokyo, Japan). For the first experiment, fourteen mice were randomly divided into three groups (rubber band only, *N* = 5; PLD treatment, *N* = 4; PLD plus rubber band, *N* = 5). For the second experiment, 14 mice were randomly divided into four groups (rubber band only, *N* = 4; PLD treatment, *N* = 4; PLD plus 50 mg/kg DXZ, *N* = 3; PLD plus 250 mg/kg DXZ, *N* = 3). The PLD treatment concentration (Doxil; Baxter Japan, Tokyo, Japan; 16.5 mg/kg, 50 mg/m^2^ [15]) was set according to the clinical dosage for platinum‐resistant ovarian cancer (50 mg/m^2^) [16–20]. Power analysis indicated a sample size of three, the minimum number required for statistical significance tests. No mice were excluded from the analysis.

Mice under 4% (induction) or 2%–3% (maintenance) isoflurane inhalation anesthesia were transcardially perfused with saline. Skin tissue was collected and stored at −80°C until protein extraction. All experiments were performed strictly according to the ARRIVE guidelines and the Guidelines for Animal Experimentation of Setsunan University. The protocol was approved by the Experimental Animal Research Committee of Setsunan University (reference numbers: K23‐27 and K22‐26).

### 2.2. Determination of Rubber Band Dimensions and Elastic Force

The width, thickness, and circumference of rubber bands (Rubber Band No. 14; KOHNAN SHOJI, Osaka, Japan) were measured using a digital caliper (LFX‐40‐042; KOHNAN SHOJI) (see Table 1). Elasticity was measured using a NEWTON METER (GN‐1; NaRiKa, Tokyo, Japan).

**TABLE 1:** Rubber band dimensions (mm).

	Width	Thickness	Inner circumference
Rubber band #14	1.056 ± 0.028	1.061 ± 0.033	103.6 ± 0.518

*Note:* Measurement is shown as means ± SEM (*N* = 10).

### 2.3. Experimental Schedule

**FIGURE 1:**
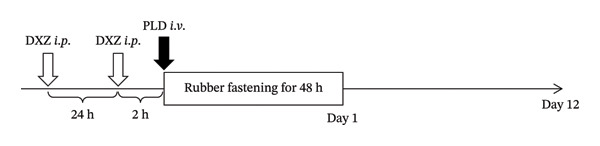
Schedule for treatment to induce HFS‐like skin damage in mice.

On the day before PLD administration, the dorsal body hair of mice was shaved under isoflurane inhalation anesthesia. Subjects were administered 5% glucose solution or PLD (16.5 mg/kg) *via* the tail vein, and the skin around the waist was gently compressed using a double‐layered rubber band for 48 h. For the second experiment (Figure 1), DXZ (AMBH303C591D; Sigma‐Aldrich Corp., St. Louis, MO, USA) [10] was administered intraperitoneally at two time points, 2 and 24 h before the PLD injection. DXZ (5 or 25 mg/mL) was dissolved in 5% dimethyl sulfoxide and then diluted in saline. Skin tissues on the back and right flank were photographed using a digital camera (X‐A5; FUJIFILM, Tokyo, Japan) to capture changes in appearance over time. Red patches were analyzed in ImageJ to calculate the skin wound area.

### 2.4. Hematoxylin–Eosin (HE) Staining

Twelve days after the end of skin compression, skin tissues from the back and right flank were excised to prepare frozen sections (10 μm). After HE staining as previously described [14], sections were photographed and histomorphometrically analyzed using an all‐in‐one fluorescence microscope (BZ‐X800; KEYENCE, Osaka, Japan). Degrees of thickening were based on the area and length of the stratum spinosum epidermis.

### 2.5. Western Blotting

Protein extracts were prepared from back and flank skin tissue, then electrophoresed on 10% or 10%–20% sodium dodecyl sulfate–polyacrylamide gels, as previously described [21]. Separated proteins were transferred onto polyvinylidene difluoride membranes (Immobilon‐P; Merck Millipore, Darmstadt, Germany). After blocking with nonspecific protein binding on the membranes was blocked using Tris‐buffered saline containing 0.1% Tween 20 (TBS‐T) and 5% skim milk (31,149‐75; Nacalai Tesque, Kyoto, Japan), membranes were incubated overnight at 4°C with rabbit anti‐phospho‐histone H2AX (Ser139) monoclonal antibody (#9718, 1:1000; Cell Signaling Technology, Danvers, MA, USA), rabbit anti‐Topo IIα polyclonal antibody (24641‐1‐AP, 1:1000; Proteintech Group Inc., Rosemont, IL, USA), rabbit anti‐Topo IIβ polyclonal antibody (A300‐950A, 1:1000; Bethyl Laboratories, Montgomery, TX, USA), rabbit anti‐β‐actin polyclonal antibody (20536‐1‐AP, 1:1000; Proteintech), or rabbit anti‐β‐actin monoclonal antibody (sc‐47778, 1:1000; Santa Cruz Biotechnology, TX, USA). The membranes were washed with TBS‐T and incubated with horseradish peroxidase‐conjugated anti‐rabbit IgG polyclonal antibody (#7074, 1:3000; Cell Signaling Technology) or anti‐mouse IgG polyclonal antibody (HAF007, 1:3000; R&D Systems, Minneapolis, MN, USA) for 1 h at 23°C. Immunoreactive protein bands were detected using a chemiluminescent substrate (Clarity Western ECL Substrate; Bio‐Rad Laboratories Inc., Hercules, CA, USA). Band intensity was measured in ImageJ and normalized to β‐actin.

### 2.6. Statistical Analysis

Data are presented as mean ± standard error (SEM). For multiple group comparisons, one‐way analysis of variance was performed, followed by Bonferroni’s or Dunnett’s multiple comparison test. Prism Version 8 (GraphPad, La Jolla, CA, USA) was used for analysis. Significance was set at *p* < 0.05.

## 3. Results

To ensure the reproducibility of the model in this study, we obtained basic data on the rubber bands via determining the relationship between band tension and folding diameter (Figure 2). Mice without and with double rubber bands had abdominal girths of 82.96 ± 1.82 and 58.17 ± 0.47 mm, respectively (*N* = 10 each).

FIGURE 2Relationship between tension and folding diameter of the rubber band used to apply pressure on the skin. Randomly selected rubber bands were used to measure the relationship between fold diameter and tension of single‐folded (a) and double‐folded (b) rubber bands. Open circles and bars represent mean ± SEM (*N* = 10), and the equations represent logarithmic approximation curves.

**Figure:**
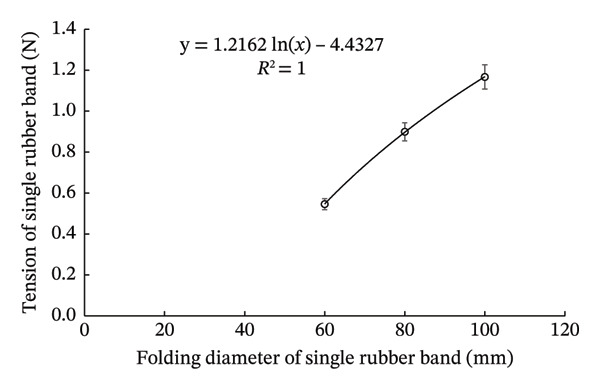
(a)

**Figure:**
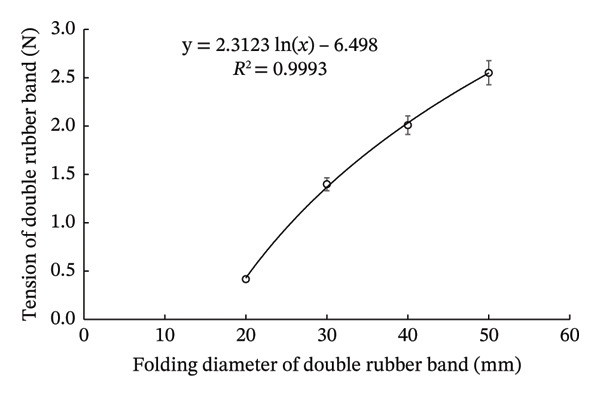
(b)

On the first day of treatment (Figure 3), we observed traces of skin compression in the control (double rubber bands only) that disappeared after Day 5 (Figure 3(a)). In the PLD‐only group, neither skin discoloration nor damage was present from Days 1–12 (Figure 3(a)). In contrast, we noted red skin in the compressed regions among mice treated with PLD plus rubber bands on Day 1; the reddened area exhibited noticeable skin damage after Day 5 (Figure 3(a)). This group also had significantly thicker epidermis in the damaged back tissue than rubber‐band‐only and PLD‐only mice (Figures 3(b), 3(c)).

FIGURE 3Effects of pressure on PLD‐induced skin damage. “Rubber band” refers to mice fitted with double rubber bands around their waist. “PLD” denotes mice administered PLD. “PLD + rubber band” describes mice subjected to both treatments. (a) Representative photographs showing the back regions of mice in groups. (b) HE‐stained images of the back skin tissue. (c) Extent of epidermal thickening. Data for each group were analyzed using Bonferroni’s multiple comparison test, and each bar represents the mean ± SEM (*N* = 4 or 5).

**Figure:**
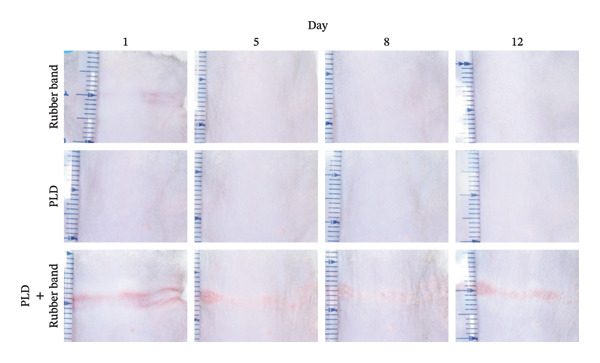
(a)

**Figure:**
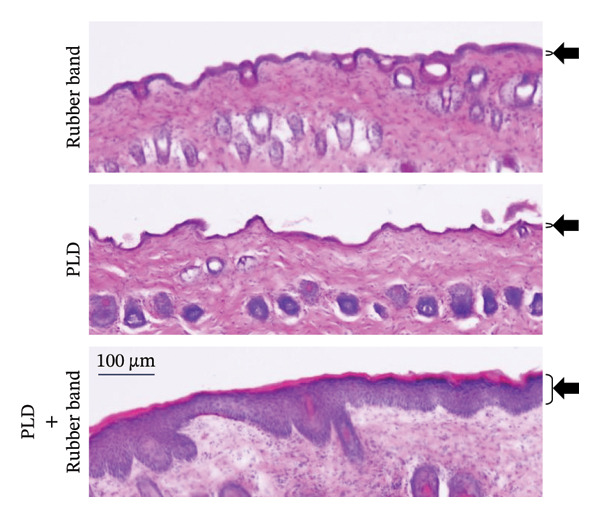
(b)

**Figure:**
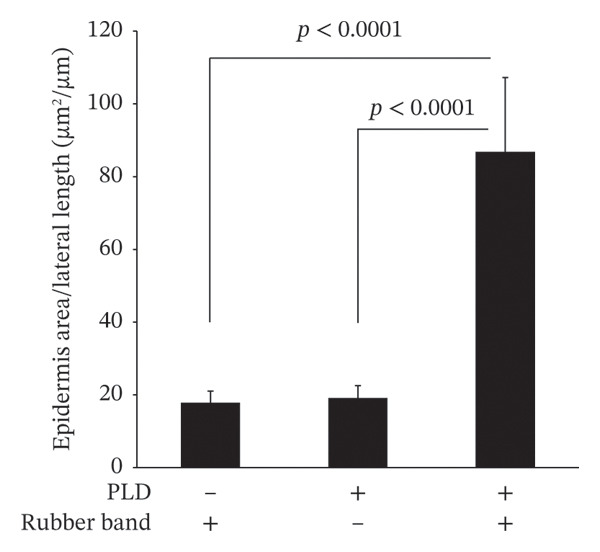
(c)

Skin compression on the back and flank areas of rubber‐band‐only control mice was observed immediately after band removal on Day 1 and disappeared after Day 5 (Figures 4(a), 4(b)). In rubber‐banded mice treated with PLD, skin damage appeared after Day 5 at sites that were visibly compressed on Day 1. Rubber‐banded mice administered DXZ + PLD exhibited less skin damage than rubber‐banded mice treated with PLD only. In addition, quantitative analysis of the wound area on the back showed no between‐group differences on Day 5; however, wound areas on Days 8 and 12 were significantly lower in the DXZ + PLD groups than in the PLD‐only group (Figure 4(c)). Results from the flank were similar to back wounds, except for the PLD‐only *vs*. DXZ (50 mg/kg) + PLD groups on Day 8 (Figure 4(d)).

FIGURE 4Wound formation and area on back and flank skin of PLD‐ and DXZ‐treated mice. (a, b) Typical changes in appearance over time of the back skin and quantitative analysis of wound area. (c, d) Typical changes in appearance over time of the flank skin and quantitative analysis of wound area. Scale bar, 5 mm. Bonferroni’s multiple comparison test was used to assess between‐group differences, and each bar represents the mean ± SEM (*N* = 3 or 4). ^∗^ *p* < 0.05; ^∗∗^ *p* < 0.01; ^§^ *p* < 0.001 (all *vs.* PLD).

**Figure:**
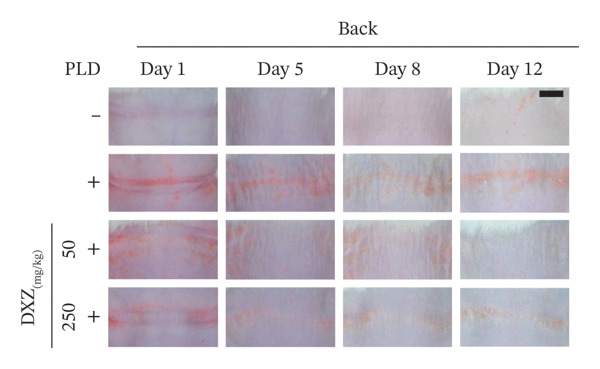
(a)

**Figure:**
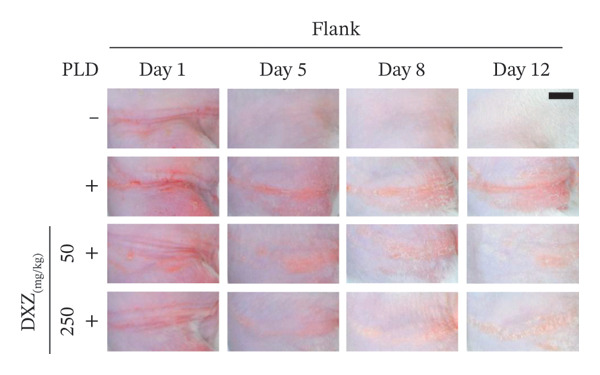
(b)

**Figure:**
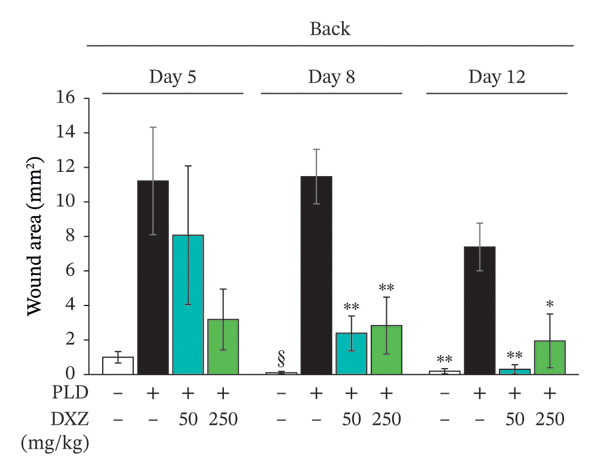
(c)

**Figure:**
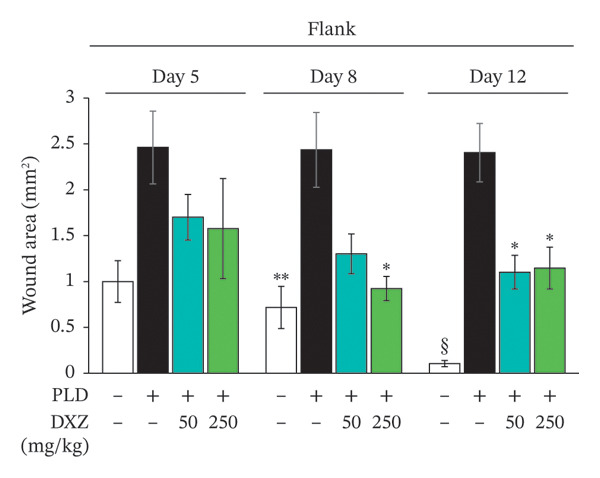
(d)

On Days 8 and 12, body weight was significantly lower in the PLD‐treated groups than in the control group (Supporting Figure 1). In contrast, the PLD and DXZ + PLD groups (50 and 250 mg/kg) did not differ.

Histomorphometry of back and flank skin sections showed that PLD‐only mice had visibly thicker epidermis than control mice, whereas DXZ + PLD mice had thinner epidermis than PLD‐only mice (Figure 5(a)). Quantitative analysis of epidermal thickening in back and flank skin tissues confirmed a significant difference between PLD and control mice (Figures 5(b) and 5(c)), as well as between both DXZ (50 and 250 mg/kg) combinations and PLD‐only mice.

FIGURE 5Thickening ratio of the spinous layer in back and flank skin of PLD‐ and DXZ‐treated mice. (a) Typical HE‐stained images of back and flank skin tissue sections on Day 12. Scale bar, 200 μm. (b, c) Quantitative analysis of the thickening ratio of the spinous layer, obtained by dividing the epidermal area by the lateral length of the skin surface. Bonferroni’s multiple comparison test was used to assess between‐group differences, and each bar represents the mean ± SEM (*N* = 3 or 4). ^∗^ *p* < 0.05; ^∗∗^ *p* < 0.01; ^§^ *p* < 0.001 (all *vs*. PLD).

**Figure:**
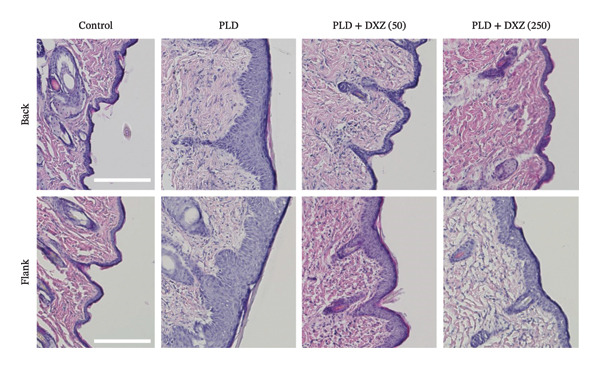
(a)

**Figure:**
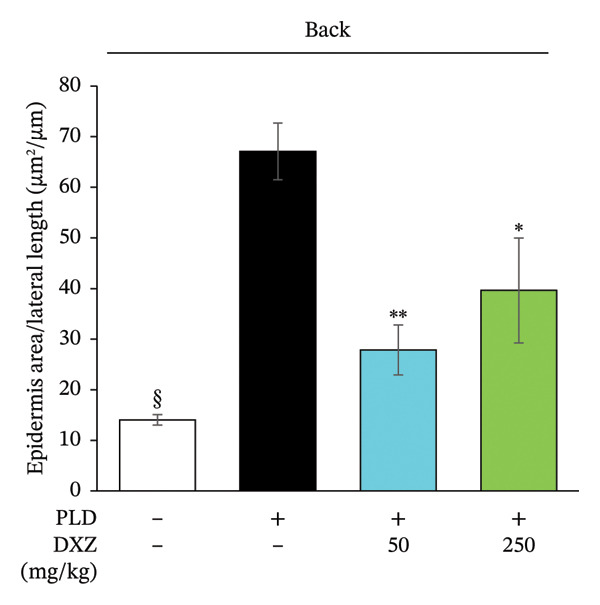
(b)

**Figure:**
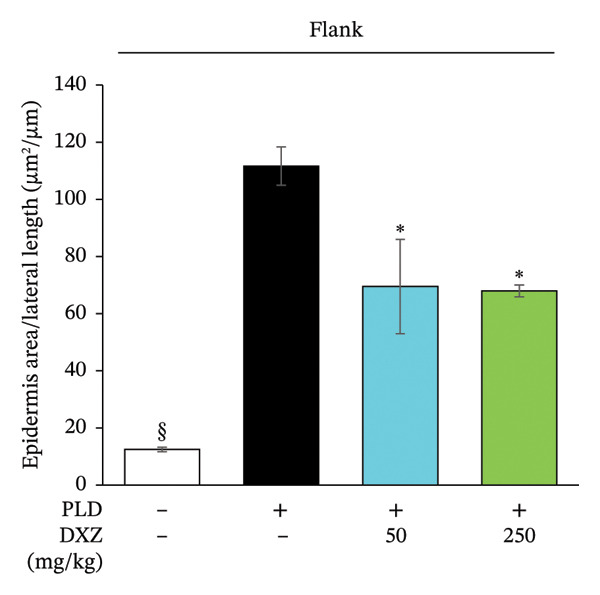
(c)

**FIGURE 6:**
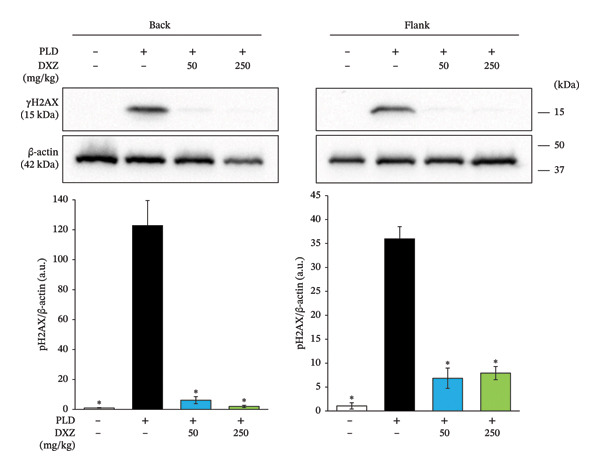
Effect of DXZ administration on phosphorylated γH2AX expression in back and flank skin tissue of PLD‐treated mice. Representative images and quantification of phosphorylated γH2AX are shown. Data were compared using Bonferroni’s multiple comparison test, and each bar represents mean ± SEM (*N* = 3 or 4). ^∗^ *p* < 0.001 (*vs*. PLD).

Phosphorylated H2AX (γH2AX) expression was evaluated as an indicator of DNA damage caused by PLD‐encapsulated doxorubicin [9, 22]. γH2AX expression significantly increased in the back and flank regions of PLD‐treated mice, whereas γH2AX was significantly downregulated in the DXZ + PLD groups compared with the PLD‐only group (Figure 6).

At 48 and 72 h postadministration, endogenous Topo IIβ expression in the heart, back, and flank regions of DXZ‐alone mice decreased from control levels (Supporting Figure 2–5). Topo IIβ expression in both the back and flank regions was significantly higher than control in the PLD‐only group and significantly lower in the DXZ + PLD groups than in the PLD‐only group (Figure 7(a)). Although Topo IIα expression showed no between‐group differences, it had a similar variation pattern as Topo IIβ (Figure 7(b)).

FIGURE 7Expression of Topo II isoforms in back and flank skin tissue of PLD‐ and DXZ‐treated mice. Representative images and quantification of Topo IIα (b) and Topo IIβ (a) expression are shown. Data were compared using Bonferroni’s multiple comparison test, and each bar represents mean ± SEM (*N* = 3 or 4). ^∗^ *p* < 0.01, ^∗∗^ *p* < 0.001 (both *vs*. PLD). Panel B *p* values were from one‐way analysis of variance.

**Figure:**
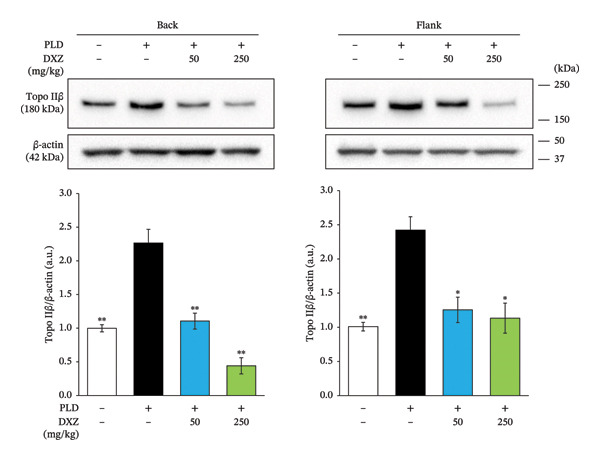
(a)

**Figure:**
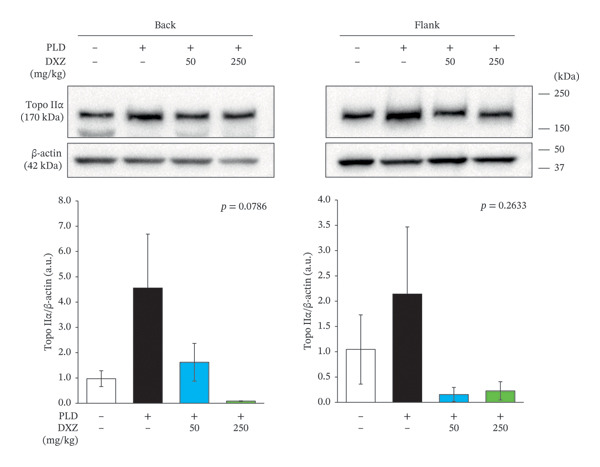
(b)

## 4. Discussion

In this study, we used rubber bands to generate a new animal model of PLD‐induced HFS‐like skin damage and applied the model to examine DXZ’s protective effects.

In PLD‐treated patients with metastatic breast cancer, HFS can develop in areas with a high frequency of exposure to pressure, including the axillary, inguinal, and sacral regions [23]. Here, we showed that a single administration of PLD resulted in HFS‐like skin damage when local pressure was applied to the skin with a rubber band. Our method needs fewer doses and less time to develop skin damage (15–12 days) than previous methods [14]. Consequently, this study may offer a simpler method for generating PLD‐induced skin damage in mice. Moreover, our technique mimics HFS pathogenesis by applying pressure to the skin.

Jaconelli et al. found mildly acanthotic epidermis in histological analyses of skin biopsies from patients with ovarian carcinoma that were treated with PLD [24]. Our findings of epidermal hyperplasia in our animal model were similar to these. Our animal model has the morphological features of PLD‐induced HFS, suggesting that the degree of epidermal thickening could serve as an objective indicator for evaluating skin disorders. In contrast, we did not find any other features of patients treated with PLD such as marked hyperkeratosis with inadequate keratosis in the stratum corneum of the epidermis, spongiosis with numerous pyknosis cells but no associated lymphocytes in the Malpighian layer, and granular layer thickening in plantar punch biopsies [25, 26]. Detailed studies are required to clarify the mechanism of PLD‐induced HFS.

Topo IIβ‐mediated DNA damage is implicated in the development of doxorubicin‐induced cardiotoxicity [9, 10, 12, 27]. Both in vivo and in vitro data show that DXZ can prevent this cardiotoxicity [8–10], with proteasome‐inhibitor experiments, demonstrating that the drug promotes Topo IIβ degradation [9, 10, 13]. However, the effect of DXZ on PLD‐induced skin damage has not been previously reported. This study found that preadministration of DXZ suppressed PLD‐induced HFS‐like skin damage. Moreover, DXZ treatment decreased Topo IIβ expression in skin tissues and simultaneously limited DNA damage. Therefore, DXZ is effective against PLD‐induced skin lesions, and the mechanism might involve downregulation of Topo IIβ expression to block DNA breakdown. Topo IIα is involved in various skin disorders, including psoriasis. Yang et al. revealed significantly upregulated Topo IIα expression in keratinocytes stimulated with psoriasis‐associated cytokines, and its knockdown suppressed cell proliferation, migration, and inflammatory responses *via* the Wnt/β‐catenin signaling pathway [28]. Zhu et al. also identified elevated Topo IIα expression in psoriatic skin lesions based on transcriptomic analyses, suggesting that it plays a role in skin pathology [29]. Bohr et al. showed that topical application of topoisomerase inhibitors such as novobiocin and nalidixic acid leads to clinical improvement in psoriatic plaques [30]. These findings collectively support the notion that Topo IIα/β plays a critical role in skin inflammation and toxicity. Therefore, the downregulated Topo IIα/β determined herein might represent a key mechanism underlying the protective effect of DXZ against HFS‐like skin damage induced by PLD. Our data also notably revealed that PLD was associated with increased expression of Topo IIβ and, to a lesser extent, Topo IIα in skin tissues. While the biological significance of this upregulation remains unclear, it might reflect a cellular response to PLD‐induced stress or damage. However, we did not investigate the upstream regulatory mechanisms involved and thus cannot draw definitive conclusions regarding the cause of the altered expression. Future studies should aim to clarify the molecular pathways through which PLD influences topoisomerase expression, as this might help to better understand the pathogenesis of HFS‐like skin damage and refine therapeutic strategies.

## 5. Conclusions

The protective effect of DXZ against PLD‐induced HFS‐like skin damage was associated with decreased Topo IIβ expression in a new mouse model of PLD‐induced HFS‐like skin damage. Further studies using this model are needed to clarify how to manage PLD‐induced HFS.

## Funding

Part of this study was financially supported by the Japan Society for the Promotion of Science KAKENHI (Grant No. JP 24K14186).

## Conflicts of Interest

The authors declare no conflicts of interest.

## Supporting Information

Additional supporting information can be found online in the Supporting Information section.

## Supporting information


**Supporting Information 1** Supporting Figure 1. Changes in mouse body weight after PLD and DXZ administration. Each bar represents mean ± SEM (*N* = 3–4). Dunnett’s multiple comparison test was applied to assess between‐group differences. ^∗^ *p* < 0.01.

**Supporting Information 2** Supporting Figure 2. Expression of endogenous Topo IIβ in back and flank skin tissues of mice. The heart was used as a positive control for Topo IIβ expression, and β‐actin was used as a loading control.

**Supporting Information 3** Supporting Figure 3. Topo IIβ expression in heart tissue of DXZ‐treated mice. Representative images (A) and quantification (B) of Topo IIβ expression.

**Supporting Information 4** Supporting Figure 4. Topo IIβ expression in the back skin tissue of DXZ‐treated mice. Representative images (A) and quantification (B) of Topo IIβ expression.

**Supporting Information 5** Supporting Figure 5. Topo IIβ expression in the flank skin tissue of DXZ‐treated mice. Representative images (A) and quantification (B) of Topo IIβ expression.

## Data Availability

The datasets used and analyzed during the present study are available from the corresponding author upon reasonable request.
