# Supplementary figures and images for: Dexrazoxane Protects Against Hand–Foot Syndrome–Like Skin Damage in Pegylated Liposomal Doxorubicin‐Treated Mice
Source: J Toxicol. 2026 Jan 30;2026:1358796. doi: 10.1155/jt/1358796 (PMC12857704; doi:10.1155/jt/1358796)

## Slide 1
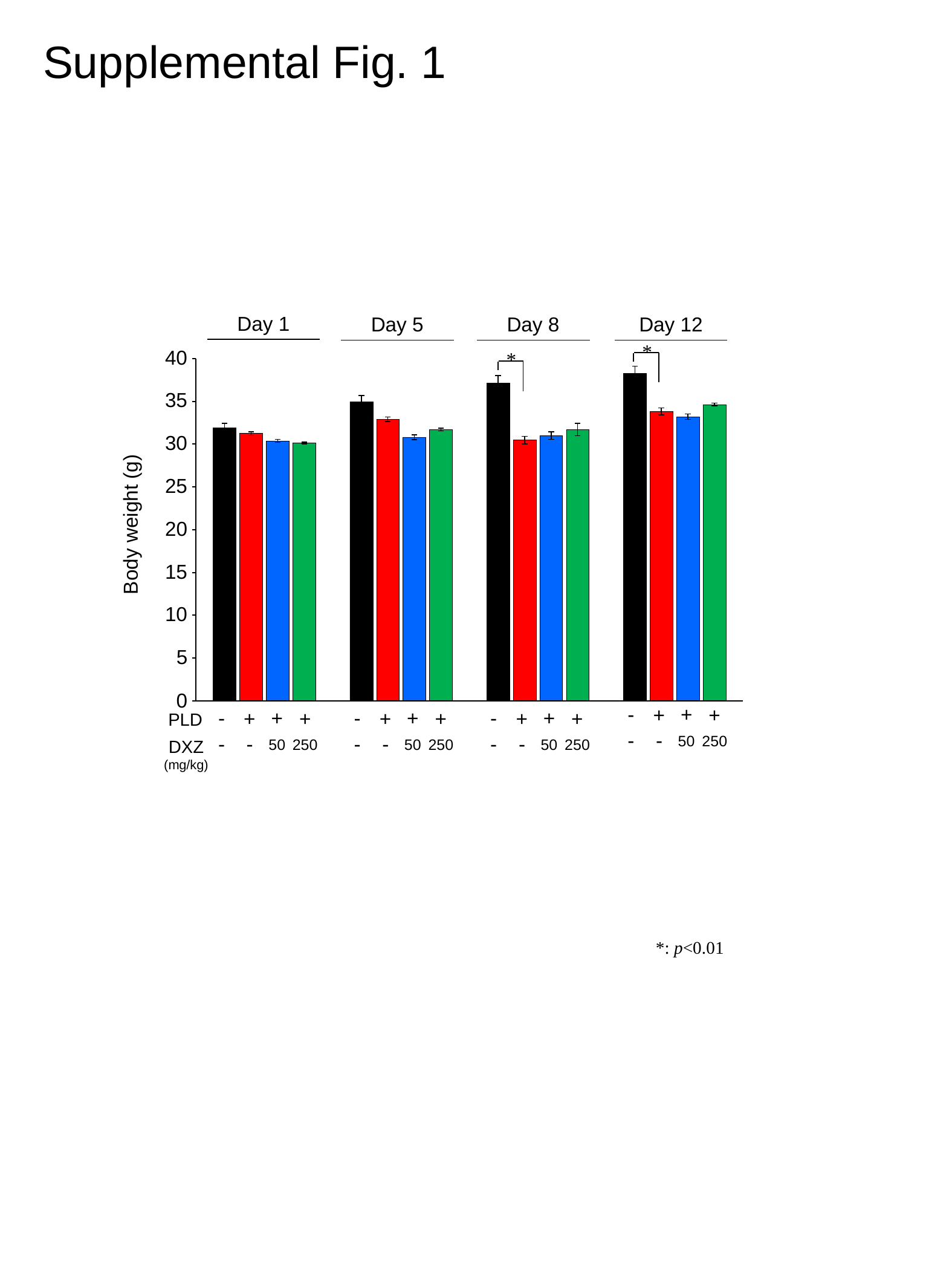

Supplemental Fig. 1
Day 1
Day 5
Day 8
Day 12
*
40
*
35
30
Body weight (g)
25
20
15
10
5
0
-
+
+
+
-
-
50
250
-
+
+
+
-
-
50
250
-
+
+
+
-
-
50
250
-
+
+
+
-
-
50
250
PLD
DXZ (mg/kg)
*: p<0.01

Supplement: Supplementary file 1 — Supporting Information 1 Supporting Figure 1. Changes in mouse body weight after PLD and DXZ administration. Each bar represents mean ± SEM (N = 3–4). Dunnett’s multiple comparison test was applied to assess between‐group differences. ∗ p < 0.01. [file JT-2026-1358796-s005.pptx]

## Slide 1
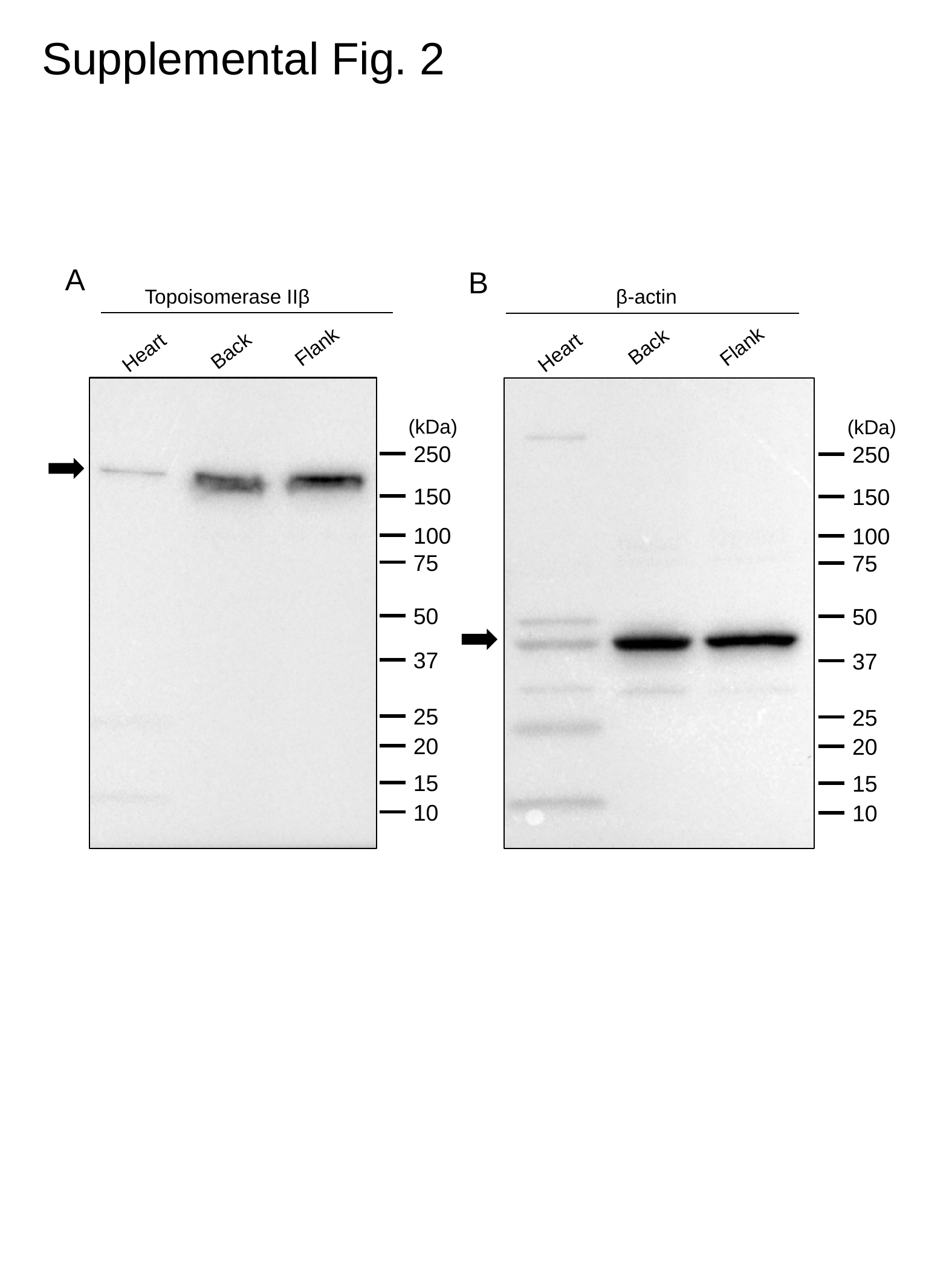

Supplemental Fig. 2
A
B
Topoisomerase IIβ
β-actin
Flank
Flank
Back
Back
Heart
Heart
(kDa)
250
150
100
75
50
37
25
20
15
10
(kDa)
250
150
100
75
50
37
25
20
15
10

Supplement: Supplementary file 2 — Supporting Information 2 Supporting Figure 2. Expression of endogenous Topo IIβ in back and flank skin tissues of mice. The heart was used as a positive control for Topo IIβ expression, and β‐actin was used as a loading control. [file JT-2026-1358796-s001.pptx]
