# Supplementary material for: Dexrazoxane Protects Against Hand–Foot Syndrome–Like Skin Damage in Pegylated Liposomal Doxorubicin‐Treated Mice
Source: J Toxicol. 2026 Jan 30;2026:1358796. doi: 10.1155/jt/1358796 (PMC12857704; doi:10.1155/jt/1358796)
Supplement: Supplementary file 4 — Supporting Information 4 Supporting Figure 4. Topo IIβ expression in the back skin tissue of DXZ‐treated mice. Representative images (A) and quantification (B) of Topo IIβ expression. [file JT-2026-1358796-s003.pptx]

## Slide 1
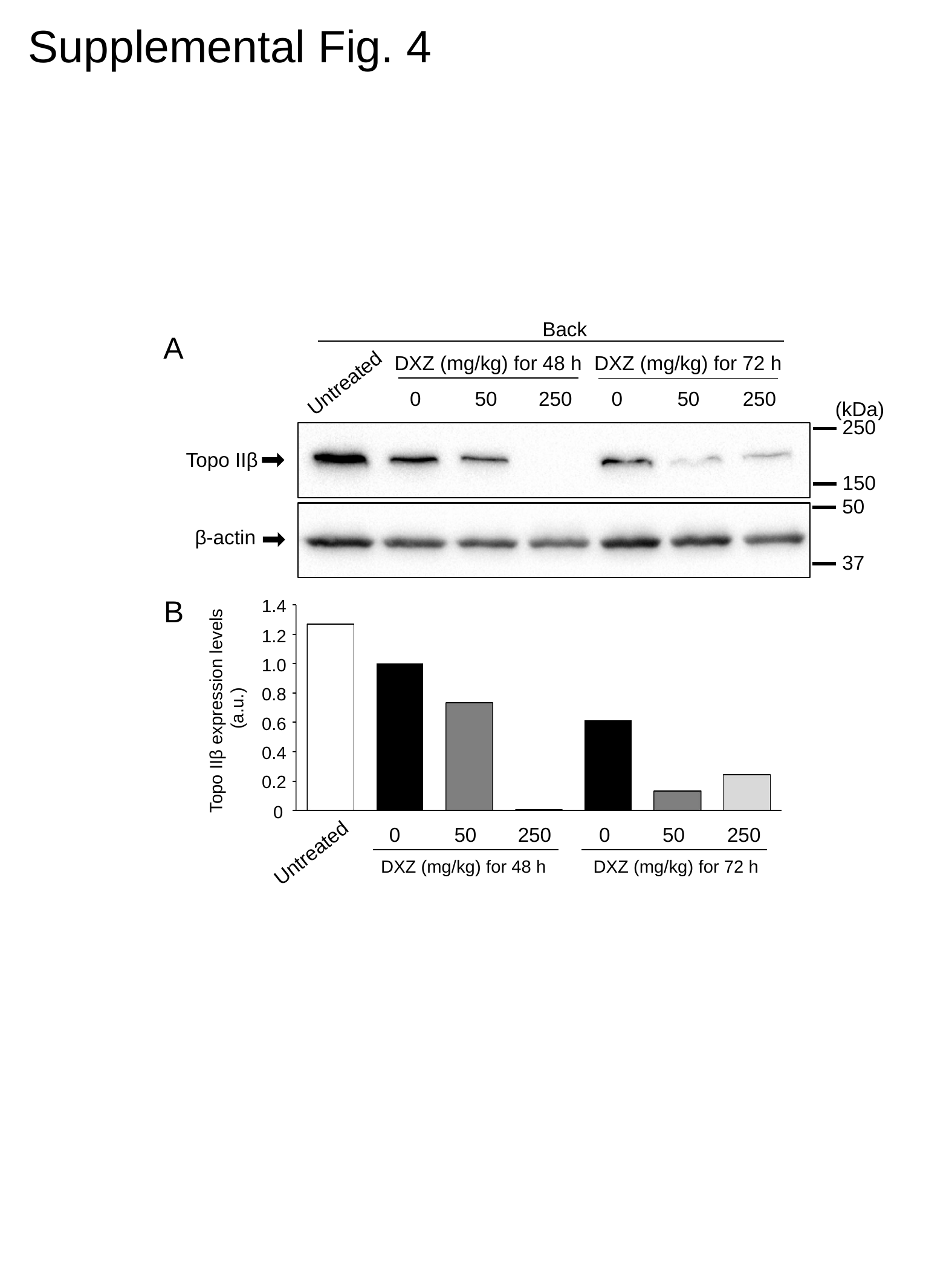

Supplemental Fig. 4
Back
A
DXZ (mg/kg) for 48 h
DXZ (mg/kg) for 72 h
Untreated
0
50
250
0
50
250
(kDa)
250
Topo IIβ
150
50
β-actin
37
B
1.4
1.2
1.0
0.8
Topo IIβ expression levels
(a.u.)
0.6
0.4
0.2
0
0
50
250
0
50
250
Untreated
DXZ (mg/kg) for 48 h
DXZ (mg/kg) for 72 h
